# Clinical Applications of Minimal Residual Disease Assessments by Tumor-Informed and Tumor-Uninformed Circulating Tumor DNA in Colorectal Cancer

**DOI:** 10.3390/cancers13184547

**Published:** 2021-09-10

**Authors:** Jun Gong, Andrew Hendifar, Alexandra Gangi, Karen Zaghiyan, Katelyn Atkins, Yosef Nasseri, Zuri Murrell, Jane C. Figueiredo, Sarah Salvy, Robert Haile, Megan Hitchins

**Affiliations:** 1 Department of Medicine, Samuel Oschin Comprehensive Cancer Institute, Cedars Sinai Medical Center, Los Angeles, CA 90048, USA; andrew.hendifar@cshs.org (A.H.); jane.figueiredo@cshs.org (J.C.F.); Sarah.Salvy@cshs.org (S.S.); robert.haile@cshs.org (R.H.); megan.hitchins@cshs.org (M.H.); 2 Department of Surgery, Samuel Oschin Comprehensive Cancer Institute, Cedars-Sinai Medical Center, Los Angeles, CA 90048, USA; alexandra.gangi@cshs.org (A.G.); Karen.zaghiyan@cshs.org (K.Z.); yosef.nasseri@cshs.org (Y.N.); Zuri.murrell@cshs.org (Z.M.); 3 Department of Radiation Oncology, Cedars Sinai Medical Center, Los Angeles, CA 90048, USA; katelyn.atkins@cshs.org

**Keywords:** circulating tumor DNA, minimal residual disease, tumor-informed, tumor-agnostic, colorectal cancer

## Abstract

**Simple Summary:**

Circulating tumor DNA, or ctDNA, are fragments of tumor DNA that can be detected in the blood of patients with colorectal cancer. Measuring ctDNA levels in the blood has shown the potential to provide important information that can be helpful in the clinical care of patients with colorectal cancer. For example, in patients with colon cancer that has been removed by surgery, measuring ctDNA in the blood can predict the likelihood of cancer recurrence, while in those with metastatic colorectal cancer, measuring ctDNA can inform the clinician whether chemotherapy is effective at earlier timepoints than currently available tests. In this review, we discuss the results from ongoing studies describing the utility of ctDNA measurements across all stages of colorectal cancer. We also discuss the various clinical scenarios that ctDNA may have the most immediate impact in colorectal cancer management.

**Abstract:**

Emerging data suggest that circulating tumor DNA (ctDNA) can detect colorectal cancer (CRC)-specific signals across both non-metastatic and metastatic settings. With the development of multiple platforms, including tumor-informed and tumor-agnostic ctDNA assays and demonstration of their provocative analytic performance to detect minimal residual disease, there are now ongoing, phase III randomized clinical trials to evaluate their role in the management paradigm of CRC. In this review, we highlight landmark studies that have formed the basis for ongoing studies on the clinically applicability of plasma ctDNA assays in resected, stage I–III CRC and metastatic CRC. We discuss clinical settings by which ctDNA may have the most immediate impact in routine clinical practice. These include the potential for ctDNA to (1) guide surveillance and intensification or de-intensification strategies of adjuvant therapy in resected, stage I–III CRC, (2) predict treatment response to neoadjuvant therapy in locally advanced rectal cancer inclusive of total neoadjuvant therapy (TNT), and (3) predict response to systemic and surgical therapies in metastatic disease. We end by considering clinical variables that can influence our ability to reliably interpret ctDNA dynamics in the clinic.

## 1. Introduction

Colorectal cancer (CRC) is the third-leading cause of cancer mortality in men and women in the U.S. with an estimated 52,980 deaths in 2021 [1]. For stage I colon cancer, surgery alone is definitive with 5-year survival rates approximating 99% [2]. Upfront surgery remains the standard for stage II colon cancer followed by observation in those with microsatellite instability-high status (MSI-H) or low-risk disease, while adjuvant capecitabine and oxaliplatin (CAPOX) for 3 months or 5-fluorouracil/leucovorin and oxaliplatin (FOLFOX) for 6 months are accepted options in those with high-risk features [2,3,4]. The standard treatment for stage III colon cancer is comprised of surgery followed by adjuvant CAPOX for 3 months in low-risk disease and CAPOX or FOLFOX for 6 months in high-risk disease [2,4,5,6]. It has been recently proposed that 3 months of adjuvant CAPOX can be used for all stage III colon cancer [5]. In locally advanced rectal cancer (LARC), neoadjuvant therapy and surgery are mainstays of treatment although total neoadjuvant therapy (TNT) has increasingly been recognized as an alternative to classical neoadjuvant chemoradiation therapy with promising clinical response rates, especially if organ preservation is preferred or in high-risk LARC [7,8,9,10].

Nearly half of all cases of CRC will develop incurable metastatic disease where the standard of care (SOC) remains systemic therapy in this setting [11]. In a subset of patients with metastatic colorectal cancer (mCRC), particularly having limited burden oligometastases within the liver or lung, systemic chemotherapy along with metastasectomy or ablative therapy carries the potential for durable long-term survival [4,9,12].

In all cases of stage I–III CRC, surveillance after surgery is routinely recommended with a combination of clinical, radiographic, endoscopic, and/or serologic (carcinoembryonic antigen or CEA) assessments over time to detect recurrences [2,4,9,13]. In mCRC, CEA has long been used in clinical practice as the standard tumor marker to assess response to systemic therapy [14]. In mCRC, monitoring of treatment responses, particularly with CEA, can assist in the prediction of response to systemic chemotherapy and prognostication of long-term outcomes [15]. Ultimately, the goal of postoperative surveillance in stage I–III CRC is to detect early relapses with the potential for curative intent resections while maximizing opportunities to improve survival in the metastatic setting.

More recently, analysis of circulating tumor DNA (ctDNA), which are fragments of DNA released by tumor cells into the bloodstream representing a fraction of the total cell-free DNA (cfDNA) pool, has become increasingly recognized as a non-invasive method to detect CRC-specific signals across both non-metastatic and metastatic settings [16]. The purpose of this review is to highlight where the field is moving in the clinical applications of ctDNA in CRC. We do not to cover the technical considerations in isolation, sample analysis, performance, validation, standardization, or bioinformatics analysis of ctDNA assays herein as these have been extensively reviewed elsewhere [16,17,18]. Specifically, we focus on the applications of ctDNA that have the most immediate potential for impact and implementation into routine clinical practice. Namely, we discuss the potential for ctDNA to (1) guide surveillance and intensification and de-intensification of adjuvant therapies in resected, stage I–III CRC, (2) predict treatment response to neoadjuvant therapy in LARC including TNT, and (3) predict response to systemic and surgical therapies in mCRC.

## 2. Detection of Minimal Residual Disease and Recurrences in Resected, Stage I–III Colorectal Cancer

Some of the first groups to demonstrate the feasibility of serial quantification of ctDNA in plasma samples from patients with CRC used mutations identified by Sanger sequencing in tumors to detect tumor-derived DNA in patient plasma by real-time PCR and BEAMing [19]. Here, ctDNA was detectable in all subjects who had predominantly metastatic disease before surgery. ctDNA appeared to be a more sensitive indicator of tumor burden than standard CEA and all subjects who had detectable ctDNA after surgery generally relapsed within 1 year. This study was among the first to support the use of ctDNA as a measure of tumor dynamics in CRC.

### 2.1. Tumor-Informed ctDNA Assessment

Since then, several groups have demonstrated that serial ctDNA assessments can detect minimal residual disease (MRD) in blood samples of patients with resected, localized colon cancer (Table 1). In a cohort of 230 resected stage II cases, ctDNA quantified by the highest mutant allele fraction (MAF) relative to the MAF in healthy control in plasma samples (Safe-SeqS assay) identified a significant difference in recurrence-free survival (RFS) among ctDNA-positive and -negative patients postoperatively [20]. Interestingly, postoperative ctDNA status had a greater impact on RFS than any individual clinicopathological risk factor or any combination of factors with a 48% sensitivity and 100% specificity of postoperative ctDNA to predict recurrence at 36 months. A median lead-time of 167 days between ctDNA detection and radiologic recurrence was observed compared to a median lead-time of 61 days between CEA elevation and radiologic recurrence (*p* = 0.04). The same Australian group later investigated the above tumor-informed ctDNA assay in a cohort of 96 stage III colon cancer patients having undergone R0 resection with blood collected a median of 42 days (interquartile range or IQR 32–52) after surgery who were planned for 24 weeks of adjuvant chemotherapy [21]. With a median follow-up of 28.9 months, postsurgical and post-adjuvant chemotherapy ctDNA-positivity was significantly associated with worse RFS compared to ctDNA-negative cases (Table 1). Again, postsurgical ctDNA-positivity was not associated with any known clinicopathologic factor, while postsurgical CEA level was elevated in 7/96 (7%) compared to 20/96 (21%) with postsurgical ctDNA-positivity, suggesting that ctDNA may be more useful to clinically guide adjuvant chemotherapy strategies than CEA. In a separate cohort of resected, stage I–III CRC patients, Safe-SeqS ctDNA plasma assays identified a 0% recurrence rate in ctDNA-negative patients suggesting the potential of ctDNA as a rule-out test for patients in whom less frequent radiographic exams or follow-ups may be sufficient [22].

The use of massively parallel, next-generation sequencing (NGS) of colorectal tumors to identify somatic mutations detected as ctDNA in plasma samples by quantitative PCR has demonstrated feasibility to detect MRD by several groups as well [31,32]. More recently, the performance of an ultradeep multiplex PCR-based NGS ctDNA assay (Signatera^TM^, Natera, Inc., San Carlos, CA, USA) using whole-exome sequencing (WES) of tumors to select for 16 high-ranked patient-specific somatic mutations has been described in a resected, stage I–III CRC cohort [24]. ctDNA was detected in 108/122 preoperative samples (88.5%), while an 88% sensitivity and 95% specificity of the plasma assay for relapse was shown over longitudinally collected samples from 75 patients. Interestingly, in 8 ctDNA-positive subjects with blood collected prior to adjuvant chemotherapy, ctDNA was not cleared in 4/8 patients (50.0%) following adjuvant chemotherapy. All of these patients experienced disease recurrence, indicating that MRD is associated with the failure to eliminate residual disease with adjuvant therapy. Of note, the Signatera test is a Clinical Laboratory Improvement Amendments (CLIA)-certified ctDNA assay that has received U.S. Food and Drug Administration (FDA) Breakthrough Device designation for MRD testing as of 8 May 2019.

Preliminary results from the CIRCULATE-Japan study, which included a prospective large-scale patient-screening registry, GALAXY, employing the Signatera ctDNA assay were recently reported [25,33]. Of 808 patients included in the analysis, 65, 280, and 301 patients had pathological stages I, II, and III CRC with preoperative ctDNA detected in 50 (77%), 267 (95%), and 288 (96%) patients, respectively. Notably, no association between *RAS*, *BRAF V600E*, and MSI status were found, but ctDNA-positivity at 4 weeks was significantly associated with an inferior DFS, suggesting that this would be an ideal time point for ctDNA-based adjuvant studies. Remarkably, the postoperative 6-month DFS in ctDNA-negative pathologic stage I–III cases was ≥99% (Table 1).

Other groups have similarly corroborated a low recurrence risk in ctDNA-negative patients for resected stage II–III CRC (2-year RFS rate of 89.4%) based on blood draws as early as 3–7 days postoperatively [26], whereas postoperative ctDNA-positivity has been associated with increased recurrence risk using tumor-informed assays in localized, resected CRC [23,34,35,36,37,38,39].

### 2.2. Tumor-Agnostic ctDNA Assessment

Beyond tumor-informed or NGS-based ctDNA assessments of MRD in localized CRC, several groups have published on the viability of tumor-agnostic ctDNA assays in this arena (i.e., blood-based ctDNA assays that are not dependent a priori on tumor profiling with detection of ctDNAs based on top-ranked hits in each patient’s tumor specimen). In a large series of resected stage III colon cancer patients, ctDNA was tested for *WIF1* and *NPY* DNA methylation in pre-adjuvant chemotherapy samples that showed high concordance with NGS-based ctDNA assessment (87/95 tested samples concordant, 91.6%) and inferior 3-year DFS for ctDNA-positive patients than ctDNA-negative patients treated with 3 or 6 months of adjuvant chemotherapy [29].

A separate group evaluated the feasibility of tumor-uninformed MRD detection using the plasma-only ctDNA assay (Guardant Reveal^TM^, Guardant Health) in a cohort of stage I–IV CRC patients undergoing curative intent surgeries [30]. Here, the ctDNA assay was designed to look for a combination of aberrant DNA methylation and targeted NGS of standard genomic alterations employed by most MRD assays without requiring parallel assessment of tumor tissue in blood samples collected at a landmark of 1 month following definitive therapy. A 100% recurrence rate was seen for those who were ctDNA-positive compared to a 24.5% recurrence rate for ctDNA-negative on landmark analysis. Sensitivity and specificity were comparable to other tumor-informed approaches (Table 1). Across all ctDNA-positive samples, 47% were positive by both epigenomic and genomic calls, while they were individually positive in 28% and 25% of samples, respectively, whereby incorporating epigenomic signatures increased sensitivity by 25–36% over genomic alterations alone.

The COLVERA^®^ (Clinical Genomics, Bridgewater, NJ, USA) assay is a 2-gene (*BCAT1*/*IKZF1*) methylation-specific, plasma ctDNA platform that is also commercially available and has shown greater sensitivity for recurrence in resected, localized CRC than CEA [28,40,41,42,43]. Among the earlier genes whose methylation in ctDNA has been described in CRC includes *SEPT9*, for which several groups have shown that postoperative ctDNA-positivity for *SEPT9* carries high specificity for predicting recurrent CRC [44,45,46,47,48] while conferring a median lead time of 8 months prior to radiographic detection of recurrence in resected CRC patients [27].

### 2.3. Interventional Clinical Trials with Blood-Based ctDNA Assays in Resected CRC

With the impressive potential to detect MRD in resected, stage I–III colon cancer through a non-invasive fashion, the logical next step in integrating these ctDNA assays into clinical practice would be to study their utility in prospective, ideally randomized control trials comparing ctDNA-informed approaches to SOC approaches. One area of potential clinical impact for ctDNA would be in the postoperative or adjuvant chemotherapy settings where survival benefits have been demonstrated for 3 or 6 months of adjuvant chemotherapy and are now widely recommended by consensus guidelines [2,4]. In the Signatera NGS-based ctDNA cohort, all patients with postoperative colon cancer with ctDNA-positivity prior to adjuvant chemotherapy and whose ctDNA remained positive after adjuvant chemotherapy experienced disease recurrence, underscoring the importance of clearance of MRD for improved postoperative DFS [24]. Interestingly, the prognosis of ctDNA-positive cases treated with 6 months of adjuvant chemotherapy approximated that of the ctDNA-negative cases treated with 3 months of adjuvant therapy in a recent cohort of resected, stage III colon cancer patients were evaluated using a tumor-uninformed ctDNA assay [29]. The worst outcomes were seen in those with ctDNA-positive high-risk stage III cases treated with 3 months of adjuvant chemotherapy, suggesting that a longer adjuvant treatment period may decrease the prognostic impact of ctDNA by clearing MRD.

Accordingly, the field is now moving towards the investigation of blood-based ctDNA assays in guiding the intensification or de-intensification of adjuvant chemotherapy in those with resected, localized colon cancer (Table 2). For example, in resected, stage II colon cancer where adjuvant chemotherapy (3 months of CAPOX) has largely been recommended in those with high-risk disease [3], several groups are seeking to investigate the escalation to 6 months of oxaliplatin-based chemotherapy (NRG-GI005 or COBRA) for stage IIA disease as well as other stage II colon cancer cohorts (CIRCULATE-PRODIGE 70, IMPROVE-IT, MEDOCC-CrEATE) who have ctDNA-positivity postoperatively. Other groups allow an intermediate 3 months of oxaliplatin-based chemotherapy for postoperative ctDNA-positive, stage II colon cancer (DYNAMIC, CIRCULATE AIO-KRK 0217). The phase II IMPROVE-IT trial is also investigating the benefit of 6 months of oxaliplatin-based chemotherapy in postoperative ctDNA-positive, stage I–II colon cancer patients who otherwise would not be receiving adjuvant chemotherapy under standard conditions. In high-risk stage II or stage III where questions of 3 vs. 6 months of oxaliplatin-based chemotherapy remain, the PEGASUS trial will uniquely investigate de-escalation strategies based on ctDNA clearance to initial adjuvant chemotherapy, while escalation to a full 6 months of systemic therapy with 5-FU and irinotecan (FOLFIRI) occurs if ctDNA-positivity remains or reappears after an initial period of clearance. In a similar population, the DYNAMIC-III phase II/III trial will be assigning patients based on ctDNA assessments to escalation of adjuvant chemotherapy (to even a triplet of 5-FU, oxaliplatin, and irinotecan or FOLFOXIRI) for ctDNA-positivity or de-escalation to no adjuvant chemotherapy or fluoropyrimidine with or without oxaliplatin for ctDNA-negativity depending on clinician discretion (Table 2). Other groups are investigating intensification of therapy with novel agents (immunotherapy), single-agent cytotoxics such as TAS-102, or combination cytotoxics (FOLFIRI, TAS-102 and irinotecan, or continuation of FOLFOX or CAPOX up to 6 months) for failure to clear ctDNA after standard adjuvant chemotherapy in high-risk stage II or stage III colon cancer.

Lastly several groups are investigating the benefit of intensified surveillance with more frequent imaging or positron emission tomography/computed tomography (PET/CT) imaging (IMPROVE-IT, IMPROVE-IT2) in those with postoperative, ctDNA-positive stage I–III colon cancers. In short, prospective, randomized trials are rapidly growing in number seeking to elucidate the clinical benefit of ctDNA-informed management approaches across a multitude of treatment settings in resected, stage I–III colon cancer. However, several questions remain on the role of ctDNA assessments in this population (see Future Considerations), and there is hope that many will be answered with results that are anticipated from these prospective studies.

## 3. Prediction of Response to Neoadjuvant Therapy in Locally Advanced Rectal Cancer

Historically, the treatment of stage II–III rectal cancer has involved a paradigm of neoadjuvant chemoradiation followed by total mesorectal excision (TME) and 4 months of adjuvant fluoropyrimidine-based chemotherapy [9,13]. However, recent strategies have employed a total neoadjuvant approach, known as total neoadjuvant therapy (TNT), which shift the adjuvant chemotherapy following TME to the neoadjuvant phase (either before or after chemoradiation or short-course radiotherapy), ensuring that all systemic therapies are completed prior to surgery. This approach is a standard option by national guidelines and is the preferred approach for high-risk LARC [9]. In either neoadjuvant approach, watchful waiting (WW) or organ preservation strategies in LARC are increasingly being investigated given that complete response (CR) rates approach 30% following neoadjuvant therapy [49,50]. While data in the WW setting require further maturation, TNT is generally the preferred approach if organ preservation is desired [7,8,9,10]. Not surprisingly, several groups have explored the role of ctDNA in predicting responses to neoadjuvant and surgical therapy in LARC to potentially assist in personalization of LARC treatments (Table 3).

### 3.1. Neoadjuvant Long-Course Chemoradiation

Among early studies, elevated baseline cfDNA has demonstrated feasibility (albeit total cfDNA and not ctDNA) to predict for tumor response or recurrence risk following neoadjuvant long-course chemoradiation in LARC [59,60]. Several other groups have shown an association between pre-chemoradiation ctDNA status and recurrence risk in LARC as well [52,61].

Using a tumor-informed ctDNA assay with blood samples collected prior to neoadjuvant long-course chemoradiation and following completion of neoadjuvant chemoradiation and surgery for LARC, no significant association was observed between pretreatment or post-chemoradiation ctDNA status and any clinicopathological factors or pathologic complete response (pCR) [51]. However, postoperative ctDNA-positivity was strongly predictive of recurrence irrespective of adjuvant chemotherapy, pathologic low risk (pCR, ypT0–2, ypN0), or pathologic high-risk (ypT3–4, ypN+) disease. Interestingly, 5 cases with an elevated postoperative CEA also had detectable postoperative ctDNA, but only 5/11 (45%) with postoperative ctDNA-positivity had an elevated CEA postoperatively suggesting that postoperative ctDNA assessment adds significant prognostic value to LARC patients with a non-elevated postoperative CEA.

In a smaller series, preoperative ctDNA assessments in patients treated with standard neoadjuvant long-course chemoradiation followed by TME did not demonstrate a significant relationship between preoperative ctDNA and pCR, although the correlation was demonstrated between preoperative ctDNA and composite end point markers of surgical outcomes [55]. Those with postoperative ctDNA-positivity had significantly worse progression-free survival (PFS) than those with negative ctDNA postoperatively (hazard ratio or HR 11.56, *p* = 0.007) although none with postoperative ctDNA MRD received adjuvant chemotherapy in this cohort.

Several groups have shown that postoperative or post-chemoradiation ctDNA status appears to be most predictive of treatment outcomes in LARC, as neither baseline nor during chemoradiation ctDNA detection showed significant correlation with any parameters that conventionally reflect tumor response [53,56,57]. One group, however, showed that patients who had persistently detectable ctDNA during the neoadjuvant period were found to have a significantly shorter metastasis-free survival [53]. In this study, 15 patients were managed on WW after neoadjuvant therapy and 10 developed local regrowth at a median of 11 months from neoadjuvant chemoradiation. Of these 10 patients, plasma was available a median of 4.5 days from local regrowth, and ctDNA was only detectable in 1 case of regrowth. Notably, variants originally tracked in plasma persisted in the regrowth tissue at a sufficiently high MAF in all these patients. When pre-neoadjuvant ctDNA-positivity is prognostic of worse overall survival (OS), it appears to be driven by a higher rate of distant metastases when compared to ctDNA-negative cases [54].

### 3.2. Total Neoadjuvant Therapy

In a cohort of 85 LARC patients (many of whom were treated with TNT) undergoing serial plasma ctDNA assessments, TNT showed a trend towards an increased tumor response (odds ratio or OR 2.9, 95% CI 0.8–12.3, *p* = 0.1178), but there was no association between pre-neoadjuvant therapy and post-neoadjuvant therapy ctDNA status and tumor response [57]. Instead, postoperative ctDNA status and change in ctDNA MAF before and after preoperative therapy remained independent prognostic factors for postoperative recurrence (Table 3).

As part of the GEMCAD 1402 phase II randomized trial enrolling high-risk LARC patients to receive TNT, 23 subjects with paired baseline and post-TNT/pre-surgery samples using a tumor-uninformed, plasma ctDNA assay showed ctDNA clearance following neoadjuvant treatment (66%) [58]. Although this was a preplanned analysis limited by small sample size, ctDNA detection was not a predictive biomarker of treatment response on the primary tumor as measured by pCR or neoadjuvant rectal score (NAR, Table 3). However, post-TNT/pre-surgery (but not baseline) ctDNA-positivity was associated with worse DFS and OS. Post-TNT/pre-surgery ctDNA was detected in 3/4 patients (75%) who developed liver metastasis compared with 4/41 patients (9.8%) who did not (*p* = 0.009), while post-TNT/pre-surgery ctDNA was undetectable in all patients that recurred to the lung only or peritoneum only. In a case series of patients with LARC treated with TNT and followed on WW due to clinical CR, detectable ctDNA levels following TNT afforded a lead time of 10 months prior to biopsy-confirmed local recurrence [62].

## 4. Prediction of Response to Systemic and Surgical Therapies in Metastatic Colorectal Cancer

The use of blood-based ctDNA assays to characterize alterations in *KRAS*, *BRAF*, *EGFR*, *HER2*, and gene fusions as resistance mechanisms to anti-epidermal growth factor receptor (EGFR) therapy and prognosticate in mCRC, particularly in colorectal liver metastases, has been well-described [63,64,65,66,67,68,69,70,71,72,73,74,75,76,77,78,79,80,81,82,83,84,85,86,87,88,89,90,91,92,93,94,95,96]. More recent ctDNA assays have been investigated in mCRC with evidence to suggest varied potential clinical applications in this space.

### 4.1. Resectable Colorectal Liver Metastases

In patients with resectable colorectal liver metastases (CRLMs), ctDNA was detectable in 46/54 (85%) patients prior to any treatment. In this group a median 40.93-fold decrease in ctDNA MAF with neoadjuvant chemotherapy was observed, although ctDNA clearance during neoadjuvant chemotherapy was not associated with a better RFS [97]. Notably, those with postoperative ctDNA-positivity had a significantly lower RFS (HR 6.3, 95% confidence interval or CI 2.58–15.2, *p* < 0.001) and OS (HR 4.2, 95% CI 1.5–11.8, *p* < 0.001) compared to patients with undetectable postoperative ctDNA, suggesting a role in guiding adjuvant therapy decisions in this arena. Ultimately, ctDNA-positivity following surgery ± adjuvant chemotherapy in this mCRC population was associated with a 5-year RFS of 0% vs. 75.6% for those with an undetectable end-of-treatment ctDNA (HR 14.9, 95% CI 4.94–44.7, *p* < 0.001).

Preoperative ctDNA status has been shown to be predictive of recurrence risk in those undergoing surgical resection for CRLMs [98,99,100,101]. Several groups have similarly shown an association between plasma ctDNA levels and tumor burden, ctDNA decrease during preoperative chemotherapy and improved tumor response, and postoperative and post-adjuvant chemotherapy ctDNA-positivity with shorter RFS after resection of CRLMs [102,103,104]. Others have similarly demonstrated the feasibility of plasma ctDNA assays to detect MRD in those with mCRC treated with neoadjuvant chemotherapy and surgically for curative intent with a potential correlation between undetectable ctDNA MRD and pCR to neoadjuvant chemotherapy [105,106].

### 4.2. Cytoreductive Surgery and Other Local Therapies

Beyond CRLMs, plasma ctDNA analyses have predicted for systemic relapse in those with mCRC undergoing cytoreductive surgery (CRS) and hyperthermic intraperitoneal chemotherapy (HIPEC) for peritoneal carcinomatosis, particularly in those with positive vs. negative ctDNAs post-HIPEC [107]. Preoperative ctDNA-positivity has shown the potential to predict for recurrence in mCRC patients undergoing CRS with or without HIPEC as well [108,109].

In mCRC patients treated with other local therapies including stereotactic ablative radiation therapy, detection of plasma ctDNA pre- and post-local therapy to oligometastatic sites was correlated with recurrence risk [110,111]

### 4.3. Response to Systemic Therapy

Early investigations in unresectable mCRC, whereby combination cytotoxic chemotherapy is standard, demonstrated feasibility in serial plasma ctDNA assessments with correlations in changes in ctDNA level with tumor response (by CEA and imaging) to various systemic therapy regimens [112,113,114,115]. Longitudinal sampling of ctDNA in this population was more sensitive than CEA for radiographic progression events where rises in ctDNA occurred significantly earlier than CEA [116]. Many groups have shown ctDNA-based genotyping of *KRAS* to have value for prognosticating survival to combination chemotherapy and predicting early emergence of resistance during chemotherapy with lead times as early as 51 days before radiographic progression [117,118,119,120,121,122,123,124].

An emerging interest in plasma ctDNA assays lies in their ability to be used as an early marker of response to systemic therapy in mCRC. Here, several groups have shown that reductions in ctDNA following initiation of chemotherapy (as early as after 1 cycle) was prognostic for improved survival and predictive of tumor response when correlated to CT responses as early as 8–10 weeks after chemotherapy initiation [125,126,127,128,129,130,131]. These findings have been concordant in the prospective PLACOL study where mCRC patients treated after cycle 1 of chemotherapy having low (≤0.1 ng/mL) plasma concentrations of methylated ctDNA experienced improved OS and higher objective response rates [132]. Interestingly, ctDNA assayed within the first 48 h of FOLFOX infusion in mCRC patients saw rapid decreases in MAFs within the first 24 h of chemotherapy. ctDNA MAFs that remained low at the time the of last blood collection (hour 52 from chemotherapy) were associated with response (stable disease or partial response) [133].

In induction strategies where mCRC patients are treated with triplet chemotherapy or alternating oxaliplatin- and irinotecan-based chemotherapy followed by maintenance fluoropyrimidine, lower ctDNA MAFs post-induction (collected ≤60 days after the 4–6-month induction) were associated with longer median PFS [134]. 

Plasma ctDNA has shown potential to correlate with radiographic response in mCRC patients treated with multikinase inhibitors and targeted therapies as well [135,136,137,138,139,140,141,142]. Lastly, ctDNA has been found to be prognostic of survival and predictive of tumor response to treatment with immunotherapy in mCRC including immune checkpoint inhibitors [143,144,145,146].

## 5. Future Considerations

### 5.1. Scenarios for ctDNA to Potentially Change Practice

With an expanding research effort on blood-based ctDNA assays in CRC, evidence thus far supports several key scenarios with greatest potential to have immediate impact based on stage of disease. In resected, stage I–III colon cancer or following neoadjuvant therapy and while on WW in LARC, plasma ctDNA offers superior sensitivity to CEA and median lead times of up to 11 months for radiographic detection of recurrence [23,28,62]. Naturally, an enhanced ability to predict for early recurrences in this population could trigger an intensified diagnostic work-up with imaging or endoscopy justified by the potential to salvage recurrences with curative intent surgery, when possible, or to maximize systemic therapy options in the setting of recurrent, metastatic disease [2]. Several randomized controlled trials, however, have failed to demonstrate a significant reduction in OS or CRC-specific mortality with intensified surveillance inclusive of more frequent imaging and CEA testing when compared to less frequent surveillance [147,148].

Whether postoperative ctDNA-guided intensified surveillance with more frequent imaging or PET/CT imaging can affect DFS or the fraction of relapsed CRC patients receiving curative intent surgeries or local therapies will be the focus of the ongoing IMPROVE-IT and IMPROVE-IT2 trials (Table 2). Others will be conducting prospective studies in mCRC to compare the performance of ctDNA with conventional CT scans and CEA in this advanced setting (NCT01983098). Perhaps more intriguing than the ability for ctDNA-positivity to trigger early diagnostic work-up for CRC recurrence, postoperatively, is the promising performance of postoperative ctDNA-negativity as a “rule-out” test. For example, results from prospective large-scale patient-screening registries have shown postoperative 6-month DFS in ctDNA-negative pathologic stage I–III cases of ≥99% [25,33]. In this seemingly reassuring scenario, further investigation should be done to compare the long-term outcomes between a less frequent surveillance program and routine surveillance.

Beyond the possibility of ctDNA to guide postoperative surveillance intensity in resected, stage I–III colon cancer, an area gaining increased interest is the ability to escalate or de-escalate adjuvant therapy dependent on the presence or absence of ctDNA MRD. In a post-hoc analysis of the IDEA trial, ctDNA-positive cases treated with 6 months of adjuvant chemotherapy approximated that of ctDNA-negative cases treated with 3 months of adjuvant therapy with ctDNA being most prognostic for DFS in those with high-risk stage III CRC (T4 and/or N2 tumors) treated with 3 months of adjuvant chemotherapy only [29]. Various strategies to intensify adjuvant chemotherapy are now being prospectively evaluated in ongoing clinical trials (Table 2) underscored by the concept that longer periods of adjuvant therapy or intensified combination chemotherapy are likely needed in those with ctDNA-positivity to clear MRD and decrease the risk of relapse. The majority of these ctDNA trials in resected, stage I–III colon cancer incorporate DFS as their primary endpoint with OS as secondary endpoints, which will be pivotal in answering the major looming question of whether such escalation of adjuvant therapy will ultimately impact OS. It has been debated that the limitations of ctDNA-guided management including the earlier treatment for recurrent, metastatic disease ultimately lies in the lack of data supporting its ability to modify the overall disease trajectory in CRC [149].

Barring a significant compromise in survival outcomes, one could argue that de-escalation adjuvant strategies in resected, localized CRC offers an equally promising scenario to change routine practice. With grade ≥3 toxicity rates approaching 60% to 6-month adjuvant oxaliplatin-based chemotherapies [3,5,6], the ability to de-escalate adjuvant therapy to 3 months or even to no adjuvant therapy in instances of postoperative ctDNA-negativity could be clinically meaningful and improve the quality of life for many patients by sparing them from debilitating toxicities such as peripheral neuropathy. An ongoing, large prospective registry will be seeking to evaluate survival in stage II–III CRC patients treated with adjuvant versus no adjuvant chemotherapy in patients with ctDNA negative test results to provide further evidence for such approaches (NCT04264702). Importantly, several prospective, randomized trials are ongoing to investigate the non-inferiority of de-escalation of adjuvant chemotherapy in ctDNA-negative cases compared to SOC (Table 2).

In the neoadjuvant treatment of LARC, ctDNA may play a role in prognosticating survival prior to neoadjuvant chemoradiation with mixed findings regarding its ability to predict for treatment response including pCR following completion of neoadjuvant therapy (Table 3). It should be noted that evidence is still building in this population with many studies limited by small sample sizes with only one recent study including a true TNT LARC cohort. However, one common theme appears to be that post-chemoradiation ctDNA status is a critical ctDNA assessment timepoint and most strongly predictive of RFS and various tumor response parameters including tumor regression grade and pCR (Table 3). Several groups have noted that ctDNA following neoadjuvant therapy in LARC cannot differentiate between minimal and no residual disease, and instead ctDNA more specifically reflects the presence of systemic disease rather than local minimal disease [51,58]. In this regard, future investigations into the role of ctDNA in the treatment paradigm of LARC may focus on identifying those who may be adequately treated with neoadjuvant chemoradiation and TME alone vs. those who would benefit from intensified TNT upfront based on baseline or pretreatment ctDNA positivity [55]. Additionally, in those undergoing TNT with chemoradiation first, a post-chemoradiation ctDNA assessment could help guide who should receive chemotherapy rather than move straight to surgery. ctDNA could also help inform who may be able to proceed to surgery altogether in LARC, while in the postoperative space, longitudinal ctDNA assessments could help identify those at risk of recurrence and needing intensification of adjuvant therapy (similar to the scenario of resected, stage I–III colon cancer). Given the limited data in LARC thus far, larger and ideally prospective studies incorporating TNT strategies are needed to tease out the utility for ctDNA in predicting tumor response, assisting the selection of candidates for WW, and identifying patients on WW with tumor regrowth.

In metastatic settings for CRC, growing evidence suggests that ctDNA positivity pre- and post-surgery (e.g., metastasectomy for CRLMs or CRS) predicts for high-risk of recurrence. ctDNA could thus inform selection of ideal candidates for surgery as well those who could benefit from further systemic therapy either in the neoadjuvant or adjuvant setting. Postoperative ctDNA positivity could also warrant intensification of adjuvant therapy or treatment with novel systemic agents, while triggering more frequent surveillance. In those with unresectable mCRC, ctDNA assessments could guide early sequencing to more effective systemic therapies. Indeed, this is the direction where several prospective clinical trials are going in further evaluating the role of ctDNA in mCRC (Table 4). For example, the Rapid 1 Trial (NCT04786600) will evaluate the impact of sequencing of FDA-approved systemic therapies after first-line therapy based on ctDNA results with a primary endpoint of OS.

### 5.2. Tumor-Informed vs. Tumor-Uninformed ctDNA Assays

Invariably, with increasing clinical usage of plasma ctDNA assays the debate on whether tumor-informed or tumor-uninformed ctDNA assays represent the optimal ctDNA testing approach will likely continue. Several tumor-informed ctDNA assays have been developed with limits of detection that are below 0.1% MAF [150]. Accordingly, the longitudinal sensitivity and specificity for recurrence approach 88% and 98%, respectively, for tumor-informed ctDNA assays. These are well above those for CEA (69% and 64%, respectively) in the same cohort [24]. The longitudinal sensitivity and specificity for recurrence approach 69% and 91%, respectively, for a recent tumor-uninformed plasma ctDNA panel [30]. The differences in analytical sensitivity between these assays are likely due to differences inherent to the tumor-informed NGS detection of top-ranked hits that are then deployed for plasma ctDNA detection. However, one can argue that the invasiveness and healthcare costs of biopsies to ascertain tumor tissue or the need for sufficient tumor tissue to be available to tailor tumor-informed ctDNA assays is countered by the noninvasiveness and convenience of tumor-agnostic plasma ctDNA assays, which do not require existing tumor tissues. Additionally, the tumor-informed assays tend to require longer and more labor-intensive processing, whereas tumor-agnostic assays offer a rapid turnaround time and are less costly, given their independence from tumor-to-plasma processing. Tumor-agnostic methylation ctDNA assays have been shown to be 83–92% concordant with NGS-based ctDNA assays, although the performance of methylation assays is likely to be improved with the development of larger panels of methylation markers [27,29]. For now, decisions on which ctDNA platform to use is likely to be supported by level of evidence from ongoing randomized clinical trials and quality of prospective, validation studies using either approach, provider preference, ease of access and familiarity of healthcare teams in requesting a specific assay, cost effectiveness as well as financial assistance to patients, and availability of tumor tissue, among other things.

### 5.3. Improving Risk Stratification through ctDNA

CEA represents the only conventional and recognized blood-based test for monitoring recurrence and response to systemic therapy in CRC. The superiority of plasma ctDNA assays over CEA are quite evident, with even more applicability in the up to 34% of CEA non-producers in patients with CRC [151]. Furthermore, several groups have shown that the prognostic potential of ctDNA is independent of known clinicopathologic risk factors. ctDNA also has a greater impact on RFS than any individual clinicopathological risk factor or any combination of factors known to predict for recurrence in CRC [20,21,24,51]. The addition of postoperative ctDNA to conventional variables including tumor differentiation, T stage, N stage, lymphovascular invasion, and post-surgery CEA has been shown to significantly improve the accuracy of recurrence prediction in resected, non-metastatic CRC [150]. Therefore, it could be proposed that ctDNA status represents an independent risk factor for recurrence from TNM staging that could be readily implemented with standard clinical covariates to improve CRC risk stratification.

There may be complementarity between methylation and NGS-based ctDNA assays to further improve upon the sensitivity of ctDNA assays as well. This was recently demonstrated in a prospective CRC cohort whereby incorporating methylation or epigenomic signatures increased the sensitivity of the ctDNA assay by 25–36% over targeted genomic alterations alone [30]. Specifically, across all ctDNA-positive samples, 28% and 25% of samples were individually positive by methylation and NGS-based testing, respectively, while 47% were positive by both epigenomic and genomic measures.

### 5.4. Timing, Clinical Scenarios That Could Impact ctDNA Shedding, and Other Considerations

Although it has been well described that ctDNA analysis in plasma samples minimizes the dilution of tumor-derived DNA and optimizes the sensitivity of ctDNA analysis over serum, a question remains as to the optimal timing of the blood collection for ctDNA assessment [152,153]. It has been shown that preoperative ctDNA positivity is predictive of recurrence risk compared to preoperative ctDNA negativity in CRC patients with both localized and metastatic disease [26,154,155]. However, most localized CRC cases are preoperative ctDNA positive (up to 90%) [27], and the constellation of evidence supports that postoperative ctDNA in resected, stage I–III CRC is most predictive of RFS (Table 1). Therefore, the most likely critical timepoint of ctDNA assessment in this setting would be the postoperative collection. In mCRC, ctDNA status following metastasectomy or radical surgery is also recognized as a pivotal timepoint of ctDNA assessment and is the focus of ongoing clinical trials exploring intensified systemic therapies (Table 4). In the neoadjuvant treatment of LARC, chemoradiation induces a strong reduction and even complete elimination of ctDNA by the end of the first week, while ctDNA levels were reduced regardless of eventual outcome [52]. A better understanding of the temporal dynamics of ctDNA release and clearance may be essential for the long-awaited applicability of ctDNA to predict for treatment response in the neoadjuvant treatment of LARC. Here, limited but growing evidence suggests that post-neoadjuvant therapy (including TNT) and postoperative ctDNA status remains the best predictor of clinical outcomes in LARC (Table 3).

Surgical trauma can induce an elevation in ctDNA levels that can persist up to 4 weeks [156]. Therefore, a postoperative blood draw for ctDNA about 4 weeks after surgery has been recommended along with a second repeat collection shortly after to mitigate the impact of trauma-induced cfDNA on ctDNA detection, especially for patients initially ctDNA negative preoperatively. Indeed, week 4 postoperative appears to be the recognized ideal timepoint of blood collection for stage I-III CRC, with several ongoing randomized clinical trials designed to collect blood for ctDNA status at weeks 4–6 postoperatively (Table 2). Stent placements, for example in those with malignant bowel obstructions, have also shown to increase plasma levels of ctDNA and would also need to be considered in the CRC population when performing ctDNA assessments [157]. In short, one can envision that preoperative ctDNA would be important to confirm the ctDNA status, but the postoperative ctDNA assessment would be most informative for assessment of MRD, prediction of recurrence risk, and tailoring of postoperative interventions in non-metastatic CRC.

Lastly, in advanced-stage tumors (including CRC), the amount of ctDNA is dependent on tumor type and dynamics of tumor size, but variables including physical exercise, time of the day, and recent meal do not significantly influence ctDNA content [158]. In mCRC patients on systemic therapy, it should be noted that ctDNA dynamics can be rapid with rapid decreases in MAFs as early as the first 48 h of FOLFOX infusion with potential correlation between ctDNA MAFs that remain low and tumor response (stable disease or partial response) [133]. ctDNA shedding and therefore detection in plasma has also been shown to be affected by site of metastases in mCRC, with the liver being among the sites of highest ctDNA shedding, while higher ctDNA content increases with CRC tumor stage [18,159]. These are clinical variables that would need to be considered in our interpretation of ctDNA results during this period of rapid clinical development.

## 6. Conclusions

Investigations into the clinical applicability of plasma ctDNA assays are rapidly underway in nearly all stages of CRC. In resected, stage I-III CRC, ctDNA assessments afford the ability to guide surveillance while identifying potential candidates for escalated or de-escalated adjuvant therapy strategies. In LARC, ctDNA assessments may predict for treatment responses to neoadjuvant therapy with growing interest into post-TNT tumor responses. In advanced disease, prediction of response to systemic and surgical therapies remains an active area of study for incorporation of ctDNA assays into the mCRC treatment paradigm. However, before plasma ctDNA assays can be implemented into routine clinical practice, a critical evaluation of their potential to meaningfully impact clinical outcomes in patients with CRC is warranted. Other clinical considerations including timing of ctDNA assessments, performance of tumor-informed vs. tumor-uninformed assays, and benefits beyond survival including quality of life and the opportunity to de-escalate chemotherapy will all need to be taken into context when integrating ctDNA into the clinic. Lastly, results from ongoing, prospective, and randomized controlled trials of ctDNA in CRC are eagerly anticipated and will hopefully shed light on these important unanswered questions.

## Figures and Tables

**Table 1:** Select landmark studies in ctDNA detection of recurrent CRC.

Setting, *n*	Tumor-Informed or -Agnostic	Key Findings	Ref.
Stage II, *n* = 230	Informed (1 tumor-derived somatic mutation with highest MAF)	3-yr RFS 0% (ctDNA+) vs. 90% (ctDNA−, HR 18, 95% CI 7.9–40, *p* = 2.6 × 10^−12^) postoperatively in those not receiving ACT; poorer RFS for ctDNA+ after ≥3 mos ACT (HR 11, 95% CI 1.8–68, *p* = 0.001)	[20]
Stage III, *n* = 96	Informed (1 tumor-derived somatic mutation with highest MAF)	ctDNA+ in 10/66 (15%) pts completing 24 wks ACT; 3-yr RFS for ctDNA+ vs. ctDNA− 47% vs. 76% after surgery (HR 3.8, 95% CI 2.4–21, *p* < 0.001) and 30% vs. 77% after ACT (HR 6.8, 95% CI 11.0–157, *p* < 0.001)	[21]
Stage I–III, *n* = 94	Informed (29 somatic gene panel)	Pretreatment ctDNA significantly lower in stage I than stage II–III tumors (*p* = 0.018); median 11.5 mos lead time over radiologic relapse with ctDNA+; poorer DFS with ctDNA+ after surgery (HR 11.64, 95% CI 3.67–36.88, *p* < 0.001) and ACT (HR 10.02, 95% CI 9.202–307.3; *p* < 0.0001)	[23]
Stage I–III, *n* = 58	Informed (1 tumor-derived somatic mutation with highest MAF)	Median lead time 4 mos over radiographic relapse with ctDNA+; recurrence rate in ctDNA+ 77% (10/13 pts); recurrence rate in ctDNA over 49 mos follow-up postoperatively 0% (0/45 pts, 95% CI 0–7.9)	[22]
Stage I–III, *n* = 125	Informed (16 high-ranked patient-specific SNVs and short indels)	Postoperative 30-day ctDNA status associated with 70.0% (7/10 pts ctDNA+) vs. 11.9% (10/84 pts ctDNA−) recurrence rate (HR for RFS 7.2, 95% CI 2.7–19.0, *p* < 0.001); of 58 pts with post-ACT samples, 7/7 (100%) ctDNA+ relapsed while 7/51 (13.7%) ctDNA− relapsed; of 75 pts with longitudinal samples, ctDNA+ associated with worse RFS (HR 39.9, 95% CI 7.5–211.0, *p* < 0.001); mean lead-time from ctDNA+ to radiographic relapse 8.7 mos (range 0.8–16.5)	[24]
Stage I–III, *n* = 808	Informed (16 high-ranked patient-specific SNVs and short indels)	ctDNA+ at postoperative wk 4, 12, and 24 associated with inferior DFS (HR 46.8, sensitivity for relapse detection 93.1%); 6-month DFS rate in ctDNA− postoperatively was ≥99%	[25]
Stage II–III, *n* = 240	Informed (425-gene panel)	2-yr RFS 39.3% (HR 10.98, 95% CI 5.31–22.72, *p* < 0.001) for ctDNA+ postoperatively days 3–7; 2-yr RFS 25.0% (12/137 pts ctDNA+) vs. 87.7% (125/137 pts ctDNA−) post-ACT	[26]
Stage I–III, *n* = 82	Agnostic (10 subregions of *SEPT9* gene promoter)	73/82 pts (89.0%) preoperative ctDNA+; ctDNA+ 2 wks postoperatively associated with poorer RFS (median 288 days vs. 460 days for ctDNA−, HR 4.20, 95% CI 2.30–18.73, *p* = 0.0005); 83.3% concordance (5/6 cases) with targeted NGS for recurrence	[27]
Stage II–III, *n* = 322	Agnostic (2-gene *BCAT1*/*IKZF1* methylation panel)	Sensitivity/specificity of 63.0/91.5% (ctDNA) vs. 48.1/96.3% (CEA) in 27/322 pts with postoperative recurrence	[28]
Stage III, *n* = 1017	Agnostic (2-gene DNA methylation panel)	ctDNA+ in 140/1017 pts (13.8%) pre-ACT; 3-yr DFS pre-ACT was 66.4% (ctDNA+) vs. 76.7% (ctDNA−, HR 1.46, 95% CI 1.08–1.97, *p* = 0.015); 5-yr OS 81% (ctDNA+) vs. 87% (ctDNA−, HR 1.56, 95% CI 1.08–2.26; *p* = 0.018); ctDNA prognostic for DFS for T4 and/or N2 tumors and ACT of 3 mos only	[29]
Stage I–IV (curative intent surgery), *n* = 84	Agnostic (aberrant DNA methylation + targeted NGS of standard CRC genomic alterations)	17/70 pts (24%) ctDNA+ at 1 mo after definitive therapy; with ≥1yr follow-up, recurrence rate 100% (15/15 pts ctDNA+) vs. 24.5% (12/49 pts ctDNA−); with longitudinal surveillance, sensitivity and specificity of 69.0% and 100%, respectively	[30]

ctDNA, circulating tumor DNA; CRC, colorectal cancer; MAF, mutant allele fraction; RFS, recurrence-free survival; HR, hazard ratio; CI, confidence interval; ACT, adjuvant chemotherapy; DFS, disease-free survival; NGS, next-generation sequencing; CEA, carcinoembryonic antigen.

**Table 2:** Select prospective and interventional clinical trials of ctDNA in resected, localized CRC.

Study	Setting, Planned *n*	Design	Primary Endpoint
IMPROVE-IT (NCT03748680)	Phase II, stage I–II, *n* = 64	Randomization (1:1) of ctDNA+ pts without standard ACT indication to intensified CT chest/abdomen surveillance or 6 mos of FOLFOX or CAPOX + intensified surveillance	3-yr DFS
NRG-GI005 (COBRA, NCT04068103)	Phase II/III, stage IIA, *n* = 1408	Randomization to Arm I: Routine surveillance or Arm II: IC of FOLFOX or CAPOX 6 mos (ctDNA+) or routine surveillance (ctDNA-)	ctDNA clearance, RFS
DYNAMIC (ACTRN12615000381583)	Stage II, *n* = 450	Randomization (2:1) to Arm A: IC of FOLFOX or CAPOX 3–6 mos (ctDNA+) or routine surveillance (ctDNA−) or Arm B: Clinician discretion (ctDNA results provided at 6 mos)	Number of pts treated with ACT, RFS
CIRCULATE-PRODIGE 70 (NCT04120701)	Phase III, stage II, *n* = 1980	Randomization (2:1) of ctDNA+ pts (T4b excluded) to FOLFOX 6 mos or observation	3-yr DFS
CIRCULATE AIO-KRK-0217 (NCT04089631)	Phase III, stage II, *n* = 4812	ctDNA−: randomization (1:4) to study surveillance or routine surveillance ctDNA+ and no MSI-H: randomization (2:1) to ACT (IC of capecitabine 6 mos or CAPOX 3 or 6 mos) or observation	DFS
MEDOCC-CrEATE (NL6281/NTR6455)	Phase III, stage II, *n* = 1320	ctDNA+ without standard ACT indication: randomization (1:1) to CAPOX 6 mos or routine surveillance ctDNA−: Routine surveillance	% of pts accepting ACT when ctDNA+
NCT04589468	Phase Ia/b, Stage II-III, *n* = 60	ctDNA+ postoperatively, enrolled to increased durations of exercise 3–6 times weekly up to 18 mos	Recommended phase II dose
IMPROVE-IT2 (NCT04084249)	Stage II high-risk/stage III, *n* = 254	ctDNA analysis every 4 mos postoperatively with randomization to intensified surveillance with PET/CT and colonoscopy at time of first ctDNA+ then PET/CT every 3 mos or standard surveillance	Fraction of relapsed pts receiving intended curative resection or local treatment
VEGA (jRCT1031200006)	Phase III, stage II high-risk/stage III, *n* = 1240	ctDNA+ within 4 wks preoperatively and ctDNA− at 4 wks postoperatively, randomized (1:1) to observation or CAPOX 3 mos (no MSI-H)	DFS
PEGASUS (NCT04259944)	Phase II, stage II high-risk/stage III, *n* = 140	Enrolled into molecular adjuvant therapy: CAPOX 3 mos (ctDNA+) or capecitabine 6 mos (ctDNA−, switched to CAPOX if ctDNA+ 1 mo later); then enrolled into molecular metastatic therapy: FOLFIRI 6 mos if ctDNA+/+, CAPOX 6 mos if ctDNA−/+ (switched to FOLFIRI if ctDNA+ after 3 mos), or de-escalation to capecitabine 3 mos if ctDNA+/− (switched to FOLFIRI if ctDNA+ after 3 mos) or surveillance if ctDNA−/− (switched to CAPOX if ctDNA+)	Number of post-surgery and post-adjuvant false negative cases after a double ctDNA-negative detection
NCT04486378	Phase II, stage II high-risk/stage III, *n* = 201	ctDNA+ prior to ACT and after SOC ACT, randomized to RO7198457 up to 12 mos or routine surveillance	DFS
NCT04920032	Phase II, stage II high-risk/stage III, *n* = 22	ctDNA+ after 3 mos of standard ACT, randomized to TASIRI or continuation of FOLFOX or CAPOX up to 6 mos	% ctDNA+ at 6 mos of ACT
DYNAMIC-Rectal (ACTRN12617001560381)	Stage II–III rectal, *n* = 408	After neoadjuvant chemoradiation and surgery, randomization to ctDNA-informed arm: Decision on 4 mos fluoropyrimidine ± oxaliplatin based on ctDNA+ or ctDNA− or SOC arm: 4 mos fluoropyrimidine ± oxaliplatin based on standard pathology risk assessment	Number of pts treated with ACT
DYNAMIC-III (ACTRN12617001566325)	Phase II/III, stage III, *n* = 1000	Randomization (1:1) to Arm A: SOC or Arm B: ctDNA-informed de-escalation or escalation based on IC SOC choice - SOC no chemo: no ACT (ctDNA−) or 3 mos fluoropyrimidine alone (ctDNA+) SOC 6 mos fluoropyrimidine alone: no ACT or 3 mos fluoropyrimidine alone (ctDNA−) or 6 mos fluoropyrimidine + oxaliplatin (ctDNA+) SOC 3 mos fluoropyrimidine + oxaliplatin: fluoropyrimidine alone (ctDNA−) or 6 mos fluoropyrimidine + oxaliplatin or 6 cycles FOLFOXIRI followed by further treatment per IC (ctDNA+) SOC 6 mos fluoropyrimidine + oxaliplatin: fluoropyrimidine alone or 3 mos fluoropyrimidine + oxaliplatin (ctDNA−) or 6 cycles FOLFOXIRI followed by further treatment per IC (ctDNA+)	Non-inferiority in 3-yr RFS of de-escalation to SOC (ctDNA-) and superiority in 2-yr RFS of escalation to SOC (ctDNA+)
SU2C (NCT03803553)	Phase III, stage III, *n* = 500	After standard ACT, randomization to FOLFIRI 6 mos (ctDNA+), active surveillance with imaging every 3 mos for 3 yr then every 6 mos after (ctDNA+), active surveillance with imaging every 3 mos for 3 yr then every 6 mos after (ctDNA−)	DFS, ctDNA clearance
ALTAIR (NCT04457297)	Phase III, stage III, *n* = 240	After standard ACT and ctDNA+ within previous 3 mos at any time postoperatively up to 2 yr after surgery, randomization (1:1) to 6 mos TAS-102 or placebo	DFS

CRC, colorectal cancer; ctDNA, circulating tumor DNA; ACT, adjuvant chemotherapy; CT, computed tomography; FOLFOX, 5-FU and oxaliplatin; CAPOX, capecitabine and oxaliplatin; DFS, disease-free survival; IC, investigator’s choice; RFS, recurrence-free survival; MSI-H, microsatellite instability-high; PET/CT, positron emission tomography; FOLFIRI, 5-FU and irinotecan; SOC, standard-of-care; TASIRI, TAS-102, and irinotecan; FOLFOXIRI, 5-FU, oxaliplatin, and irinotecan.

**Table 3:** Studies of ctDNA prediction of treatment responses to neoadjuvant therapy in locally advanced rectal cancer.

Treatment Type, *n*	Tumor-Informed or -Agnostic	Key Findings	Ref.
Long-course CRT, *n* = 159	Informed (1 tumor-derived somatic mutation with highest MAF)	Conversion from pre-CRT ctDNA+ to ctDNA 4–6 wks post-CRT not associated with pCR (pCR vs. non-pCR, 95% vs. 88%, *p* = 0.46); no difference in RFS between pre-CRT ctDNA+ vs. ctDNA− (HR 1.1, 95% CI 0.42–3.0, *p* = 0.823); 3-yr RFS for post-CRT ctDNA+ (50%) vs. ctDNA− (85%, HR 6.6 95% CI 2.6–17, *p* < 0.001)	[51]
Long-course CRT, *n* = 36	Informed (6-gene panel selected from frequent somatic oncogenes in rectal cancer)	3-yr OS 91.2% (ctDNA−) vs. and 71.4% (ctDNA+) pre-CRT; during first wk of CRT, ctDNA eliminated or reduced from circulation in all pts; no association between change in ctDNA levels (before and during CRT) and TRG or TNM staging	[52]
Long-course CRT, *n* = 47	Informed (up to 3 tumor-derived somatic mutations with highest MAF)	Poor responders (MRI TRG 3–5) were more likely to have post-CRT ctDNA+ (9/27, 33%) than good responders (MRI TRG 1–2, 1/20, 5%, *p* = 0.03); no difference in MRI TRG response between ctDNA+ vs. ctDNA- at any other timepoint of CRT	[53]
Long-course CRT, *n* = 146	Agnostic (*NPY* DNA methylation)	Baseline ctDNA+ not associated with clinical T or N stage or TRG but associated with worse 5-yr OS (47% vs. 69%, *p* = 0.02) mainly driven in rate of distant metastases at 5 yrs (55% vs. 72%, *p* = 0.01)	[54]
Long-course CRT, *n* = 29	Informed (using ≥1 tumor-derived somatic mutation)	Based on preoperative ctDNA status, R0-NN resection rate 88% (ctDNA−, n = 17) vs. 44% (ctDNA+, n = 9, *p* = 0.028); favorable NAR-low or -intermediate scores 88% (ctDNA−) vs. 50% (ctDNA+, *p* = 0.059); pCR rate 11% (ctDNA+) vs. 24% (ctDNA−, *p* = 0.63) not significantly different	[55]
Long-course CRT, *n* = 103	Informed (using ≥1 tumor-derived somatic mutation)	Baseline ctDNA+ rate 75%, which decreased to 15.6% 2–3 wks after initiation of CRT, and decreased to 10.5% and 6.7% before and after surgery, respectively; preoperative ctDNA+ rate significantly lower in pts with favorable TRG 0–1 (*p* < 0.001), pCR (*p* = 0.02), pathologic T stage 0–2 (*p* = 0.002), and post-CRT MRI-defined clinical extramural vascular invasion negativity (*p* < 0.002)	[56]
Long-course CRT only or TNT, *n* = 85 (*n* = 39 TNT)	Agnostic (14-gene panel of commonly altered somatic mutations)	No significant association was observed between baseline, post-treatment, and postoperative ctDNA status and the rate of responders (pCR and cCR); significant association between response and change in ctDNA by MAF before and after preoperative treatment (≥80% vs. <80%, OR 8.5, 95% CI 1.4–163, *p* = 0.015)	[57]
TNT, *n* = 60	Agnostic (aberrant DNA methylation + targeted NGS of standard CRC genomic alterations)	No association between baseline ctDNA detection and pCR (*p* = 0.134); presurgery ctDNA detection not associated with pathologic ypT or ypN status (*p* = 0.8969 and *p* = 0.586, respectively; neither baseline nor presurgery ctDNA status associated with NAR score (*p* = 0.6 and *p* = 0.9, respectively)	[58]

ctDNA, circulating tumor DNA; CRT, chemoradiation; MAF, mutant allele fraction; pCR, pathologic complete response; RFS, recurrence-free survival; HR, hazard ratio; CI, confidence interval; OS, overall survival; TRG, tumor regression grade; R0-NN, margin-negative, node-negative resection rates; NAR, neoadjuvant rectal score; TNT, total neoadjuvant therapy; cCR, clinical complete response; OR, odds ratio. CRC, colorectal cancer; ACT, adjuvant chemotherapy; DFS, disease-free survival; NGS, next-generation sequencing; CEA, carcinoembryonic antigen.

**Table 4:** Select prospective and interventional clinical trials of ctDNA in advanced or metastatic CRC.

Study	Setting, Planned *n*	Design	Primary Endpoint
NCT03832569	Phase I, non-stage IV MSI-H solid tumors, *n* = 10 including CRC	4 months after SOC therapy (surgery, chemotherapy, radiation as appropriate), adjuvant pembrolizumab for ctDNA+	ctDNA clearance at 12 mos
TACT-D (NCT03844620)	Stage III–IV CRC pretreated ≥2 lines, *n* = 100	Randomization to Arm I: regorafenib or TAS-102 with ctDNA testing or Arm II: regorafenib or TAS-102 as per SOC	Early change in ctDNA as predictor of radiographic progression, TRAEs
NCT04752930	Stage IV, *n* = 138	6–12 mos after radical surgery for CRC, randomized to diagnostic laparoscopy if 2 consecutive ctDNA+ tests within 1 mo of enrollment with CRS + HIPEC for PCI < 20 or SOC surveillance	Peritoneal metastasis free survival
NCT03436563	Phase Ib/II, stage IV, *n* = 74	Cohort D (ctDNA+) after resection of all known liver metastases, single-arm M7824 immunotherapy for 6 doses (no MSI-H)	ctDNA clearance
OPTIMISE (NCT04680260)	Phase II, stage IV, *n* = 350	After radical treatment for metastatic spread (resection, radiofrequency ablation, stereotactic body radiation therapy, or other experimental local treatment options), randomized (1:1) to ACT per SOC or ctDNA-informed approach with escalation to FOLFOXIRI 4 mos then 5-FU 2 mos (ctDNA+) or de-escalation per clinical discretion (ctDNA−)	2-year recurrence free rate
Rapid 1 Trial (NCT04786600)	Phase II, stage IV, *n* = 78	After first-line systemic therapy, randomized to ctDNA-informed arm where sequencing of FDA-approved drugs based on ctDNA results and/or imaging or SOC arm where sequencing of FDA-approved drugs based on standard imaging	OS

CRC, colorectal cancer; ctDNA, circulating tumor DNA; MSI-H, microsatellite instability-high; SOC, standard-of-care; TRAEs, treatment-related adverse events; CRS, cytoreductive surgery; HIPEC, hyperthermic intraperitoneal chemotherapy; PCI, peritoneal cancer index; ACT, adjuvant chemotherapy; FOLFOXIRI, 5-FU, oxaliplatin, and irinotecan; OS, overall survival; FDA, U.S. Food and Drug Administration.
